# Assessing scaling strategies for nature-based solutions: An indicator-based analysis of policy documents in the Canary Islands

**DOI:** 10.1007/s13280-026-02360-8

**Published:** 2026-04-12

**Authors:** M. Susana Orta-Ortiz, Davide Geneletti

**Affiliations:** Planning for Ecosystem Services and Urban Sustainability Lab, Department of Civil, Environmental and Mechanical Engineering, University of Trento, Via Mesiano, 77, 38123 Trento, Italy

**Keywords:** Cities, Ecosystem services, Spatial planning, Sustainability transition, Transformative change

## Abstract

As nature-based solutions (NBS) gain momentum in global and EU agendas for climate, biodiversity, and societal challenges, a key question is how to scale them beyond isolated interventions. This study examines three complementary scaling strategies: scaling-out (spatial expansion), scaling-up (institutional adoption), and scaling-deep (cultural integration), within the Canary Islands' multi-level governance system. We conducted a qualitative content analysis of 23 policy documents, identifying 445 NBS proposals across four intervention types and 14 ecosystem-based sub-types. These proposals were assessed using 11 quantitative indicators that measure spatial distribution, governance and sectoral integration, and decision-makers' values. Findings show uneven progress across strategies. Scaling out is the most advanced. Scaling up is constrained by limited actor diversity and weak collaborations, although municipal actors show bottom-up potential. Scaling deep is least developed, with multifunctional design rare and isolated. Collaborative proposals address a few goals, limiting multifunctionality. We conclude with practical and research directions to strengthen interconnections among scaling strategies.

**Supplementary Information:**

The online version contains supplementary material available at 10.1007/s13280-026-02360-8.

## Introduction

In the last decade, the concept of nature-based solutions (NBS)—defined as “actions to protect, conserve, restore, sustainably use, and manage natural or modified terrestrial, freshwater, coastal, and marine ecosystems to address social, economic, and environmental challenges effectively and adaptively, while simultaneously providing human well-being, ecosystem services, resilience, and biodiversity benefits” (United Nations Environment Programme 2022)—has gained prominence in global and EU policy frameworks (European Environment Agency 2021). While nature has long been used to address urban challenges (Ebenezer Howard 1902; La Rosa et al. 2021), the novelty of NBS lies in its multifunctionality and a systemic view of socio-ecological-technological systems (Cohen-Shacham et al. 2019; Geneletti et al. 2020).

According to the EU Biodiversity Strategy for 2030 “[NBS] should be systematically integrated into urban planning” (European Commission 2021), while the EU Nature Restoration Regulations sets binding targets for urban green space and canopy cover (European Union 2024). Globally, the UN Decade on Ecosystem Restoration 2021–2030 emphasizes “[mainstreaming] nature-based thinking into city management and planning practices” (United Nations 2023), and the Paris Agreement’s first stocktake urges parties to “accelerate the use of ecosystem-based adaptation and nature-based solutions” (UNFCCC. Conference of the Parties serving as the meeting of the Parties to the Paris Agreement (CMA) 2023). These initiatives promote scaling strategies to amplify NBS benefits through broader implementation, policy integration, and transformative planning practices.

Scaling refers to the process through which an innovation grows and expands its influence across different scales and/or domains (Lam et al. 2020). Drawing on the social innovation literature, particularly the framework proposed by Moore et al. (2015), this paper focuses on three distinct scaling strategies as mechanisms for NBS mainstreaming (Adams et al. 2023a, b): (i) *scaling out*, impacting diverse spatial contexts, (ii) *scaling up*, impacting policy frameworks, and (iii) *scaling deep*, impacting cultural values and beliefs.

Scaling-out strategies increases implemented NBS in various contexts for long-term, large-scale benefits (Lam et al. 2020), while adapting to local specificities and challenges (Dorst et al. 2019). The Singapore ABC Waters Programme exemplifies this, with over 100 canal and stream restorations projects citywide to improve water quality and reduce flooding (Public Utilities Board 2018). In Europe, the EU Biodiversity Strategy 2030's Urban Nature Plans are a promising scaling-out instrument for NBS.

Scaling-up strategies promote NBS across governance levels and sectors, driving structural changes in planning and institutions (Moore et al. 2015). In climate policy, this corresponds to vertical and horizontal upscaling (Fuhr et al. 2018; Smeds and Acuto 2018). It occurs when collaborations foster adoption (Albert et al. 2020; Frantzeskaki 2019; Palomo et al. 2021) and when pioneering cities inspire others to adopt sustainability solutions (Lambin et al. 2020). In this sense, public, private, and civil society actors can enact policies, partnerships, and capacity-building to accelerate uptake (Frantzeskaki et al. 2020).

Scaling-deep strategies sustain NBS by reshaping people's values and lifestyles toward nature (Moore et al. 2015), involving co-learning processes, and impactful narratives that generate new cultural ideas (Bennett et al. 2016; Lam et al. 2020). Community gardens exemplify this by fostering healthy behaviors and dietary changes (Fischer et al. 2019), intercultural knowledge sharing, and mutual support in crises (Shimpo et al. 2019). In Barcelona, bottom-up garden initiatives fostered ecosystem services valorization and stewardship (Langemeyer et al. 2018).

These scaling strategies are interdependent and complementary. Scaling out in cities can generate evidence that drives scaling up to higher governance levels (Smeds and Acuto 2018), as seen in Barcelona and Vittoria where greening plans influenced national law (Kauark-Fontes et al. 2023). Regional and national policy reforms can, in turn, spur laggard cities to adopt scaling-out strategies (Fuhr et al. 2018). Moreover, scaling-out strategies can create fertile ground for scaling-deep processes, as broader exposure to NBS impacts can foster cultural transformation. Sarkki et al. (2024) suggest that the diffusion of restoration measures in the Kiiminkjoki River basin in Finland, led by local actors and involving extensive voluntary work, has created space to challenge the prevailing norms and values of land-use decision-makers.

To the best of our knowledge, only two papers have applied a multi-strategy scaling framework to NBS. Plassnig et al. (2022) explored urban agriculture scaling, albeit from a different perspective (that of social enterprises) compared to the sustainability transition lens guiding our study. Sarkki et al. (2024) explored five forms of NBS scaling in river basins, providing concrete examples. Both use qualitative methods to identify key elements for successful NBS scaling strategies and interdependencies. Research on individual scaling strategies is more common. For example, scaling-out studies have developed assessment methods, tools, and databases to enhance NBS application and transferability (Raymond et al. 2017; Schröter et al. 2021; Voskamp et al. 2021; Cortinovis et al. 2022; Orta-Ortiz and Geneletti 2023; Roemer et al. 2023; Adem Esmail et al. 2025). Scaling-up research shows increasing but inconsistent NBS uptake (Cortinovis and Geneletti 2018; Baravikova 2020; Brillinger et al. 2020; Kauark-Fontes et al. 2023; De Los Casares and Ringel 2023), highlighting polycentric governance models (Zingraff-Hamed et al. 2021), multi-actor processes (Toxopeus et al. 2020; Frantzeskaki and Bush 2021), and intermediary organizations for cross-level adoption (Fastenrath et al. 2020; Frantzeskaki and Bush 2021). Scaling-deep strategies remain underexplored, been primarily investigated through studies on public perceptions of ecosystem services (Ko and Son 2018; Lopez et al. 2021) and the links to pro-environmental behaviors (Pico Parra 2024; Karimi-Malekabadi et al. 2025).

Our study investigates the three scaling strategies for NBS within a multi-level governance context. Specifically, it assesses the extent to which institutional actors and related policy documents (i) propose NBS interventions for different ecosystem types and spatial locations (*scaling out*), (ii) adopt NBS interventions across governance levels and sectors (*scaling up*), and (iii) recognize nature’s plural values (*scaling deep*). Using content analysis of regional to local policy documents across relevant sectors, we identify NBS proposals for different types of interventions (creation of new ecosystems, enhancement, management and protection of existing ones) and ecosystems, and characterize them using quantitative indicators that describe elements, such as frequency, detail, spatial scope, proponents, implementers, functions, and societal challenges. The ultimate goal is to unveil patterns in NBS scaling strategies and provide insights to inform efforts to expand the use of NBS.

Content analysis organizes textual data into theory-guided categories through coding operations to identify recurring patterns (Kohlbacher 2006). It has been widely applied to study the scaling of innovation in health (Birkholz et al. 2023), transport (Soliz et al. 2023), and circular economy (Muzamwese et al. 2024). In the NBS research, document-based content analysis has been applied to assess NBS integration into planning and policy frameworks (Cortinovis and Geneletti 2018; Baravikova 2020; Brillinger et al. 2020; Kato-huerta and Geneletti 2023; Kauark-Fontes et al. 2023), and institutional enablers and barriers to scaling (Fastenrath et al. 2020). In our study, it enables systematic assessment of policy-based evidence on NBS scaling.

The case study is represented by the Canary Islands in Spain, one of the most biodiverse regions on Earth, which currently faces anthropogenic pressures—especially from tourism development and urban growth—that threaten species and ecosystems (Fernández-Palacios and Whittaker 2008; Gómez et al. 2020; Hästbacka et al. 2024; Pérez-Hernández et al. 2020). The multi-level governance system of this region is structured into the Autonomous Community of the Canary Islands, two Provinces (Las Palmas and Santa Cruz de Tenerife, covering the eastern and western islands, respectively), seven Insular Councils (each for the seven main islands), and 88 municipalities.

## Materials and methods

### Selection of policy documents and content analysis

We focused on the municipality of Las Palmas de Gran Canaria (LPGC) as the most populated one in the region, and its related Insular Council of Gran Canaria. The analysis covers all relevant municipal, insular, and regional policy documents across sectors. Policy documents are broadly defined as official written records issued by public authorities that outlines goals, principles, and actions to guide decision-making in a specific sector, including plans, strategies, guidelines, programs, and regulations (Sedlačko 2018). To select documents, we identified the policy areas prioritized in the Canarian Agenda for Sustainable Development 2030 and linked to challenges addressable by NBS. All publicly accessible documents in these areas were downloaded from official institutional websites for use in the content analysis.

The content analysis identified and described NBS proposals, presented either as strategies or actions. Strategies refer to general goals or intentions that do not include specific, actionable measures: for example, increasing native vegetated areas or expanding tree canopy cover. Actions are concrete interventions targeting specific sites and involving clearly defined activities: for instance, planting a specified number of new trees in a designated area.

The content analysis combined both inductive and deductive approaches within the MAXQDA software (Kuckartz and Rädiker 2019). First, NBS proposals were identified inductively through keyword searches, guided by a list compiled from existing NBS classifications (Table S1 of the Supplementary Material) (Babí Almenar et al. 2021; Bell et al. 2019; Castellar et al. 2021; Strosser et al. 2015; The World Bank 2021). Keywords were run through the lexical search engine to locate relevant text fragments, which were thoroughly revised and coded according to the classification of NBS types (Table 1). Subsequently, a deductive thematic coding was applied to the sections of text where NBS proposals were identified, in order to extract all variables required for indicators calculation. The following information was systematically coded:

- NBS sub-types: inferred from revised documents, it distinguishes among the ecosystem types specific of the Canary Islands (del Arco Aguilar et al. 2010; Fernández-Palacios and Whittaker 2008).
- Proposal’s detail level: four categories are listed in Table 2.
- Spatial scale: from local to neighborhood, municipal, insular, and regional scales (Table 3).
- Actors: documents’ authors recorded as NBS proponents and other entities explicitly cited as responsible for implementation recorded as developers.
- Governance level: corresponding to the issuing authority (municipal, insular, regional).
- Functions: expressed as contributions to solving urban challenges and supplying ecosystem services. Challenges followed Babí Almenar et al. (2021) covering environmental, social, and economic aspects and spatial issues (e.g., inadequate infrastructure, lack of cohesion). Targeted ecosystem services were classified using the Common International Classification of Ecosystem Services (CICES). This information was extracted from the sections where proposals appeared and from the objectives described in the respective policy documents.

**Table 1:** Classification of NBS types, modified after The World Bank (2021) and Eggermont et al. (2015)

NBS type		Description
Type A	Creation of new ecosystems	Establishment of new and novel ecosystems (i.e., *new*: natural or semi-natural areas introduced in highly modified settings; *novel*: engineered or hybrid ecosystems such as green roofs, infiltration ponds, etc.) to recreate ecological functions where ecosystems have been lost due to urbanization or land-use change. It also includes the conversion of an ecosystem into a different type
Type B	Enhancement of ecosystems	Restoration and rehabilitation of degraded ecosystems to improve ecosystem functioning and recover native conditions
Type C	Management of ecosystem	Active management of ecosystems to optimize the sustainable use of natural resources, including agroecological practices (e.g., use of organic fertilizers) and vegetation maintenance techniques (e.g., selective clearing, density control, and mixing of species and age classes)
Type D	Protection of ecosystems	Prevention of the degradation of natural ecosystems by maintaining current protection status, extending protection to previously unprotected areas, and physical protection measures

**Table 2:** Levels of details in the description of NBS proposals, and associated scores (described in “Indicators to assess NBS scaling strategies” section)

Level of detail	Description	Scores
Low	Proposals detail one aspect among ecosystem type, location, size, or biotic/abiotic features	1
Medium	Proposals detail two aspects	2
High	Proposals detail three aspects	3
Very high	Proposals detail all four aspects	4

**Table 3:** Spatial scales in the description of the NBS proposals, and associated scores (described in “Indicators to assess NBS scaling strategies” section)

Spatial scales	Description	Scores
Local	A single intervention within a building block or small land patch	1
Neighborhood	Multiple interventions within a neighborhood	2
Municipal	Interventions distributed across a municipality	3
Insular	Interventions spanning multiple municipalities	4
Regional	Interventions covering multiple islands	5

### Indicators to assess NBS scaling strategies

We propose a set of indicators to assess the three scaling strategies (Table 4), based on information retrieved for each NBS proposal. Indicators are computed by NBS types and ecosystem-based sub-types to capture scaling insights.

**Table 4:** Scaling-out, scaling-up and scaling-deep indicators for NBS

Scaling strategies	Indicators		Purpose of the indicators
Scaling out	I.1	Total number of NBS proposals	Describe the spatial extension of the territory concerned by NBS and how advanced their design is
	I.2	The average level of detail	
	I.3	The average spatial scale	
Scaling up	I.4	Total number of NBS proponents	Describe the landscape of and collaboration between (institutional) actors involved with NBS across sectors and governance levels
	I.5	Percentage of proposals resulting from actors’ collaborations (including proponents and developers)	
	I.6a	Number of proposals with multi-level collaborations	
	I.6b	Number of multi-level collaborations	
	I.7a	Number of proposals with cross-sectoral collaborations	
	I.7b	Number of cross-sectoral collaborations	
	I.8a	Number of proposals with multi-level and cross-sectoral collaborations	
	I.8b	Number of multi-level and cross-sectoral collaborations	
Scaling deep	I.9	Total number of functions	Assess the recognition and embeddedness of NBS multifunctionality, serving as an implicit signal of an institutional cultural shift toward a broader appreciation of nature’s multiple values
	I.10	Percentage of multifunctional proposals	
	I.11	Average number of functions assigned to multifunctional proposals	

Scaling-out indicators, which include the number of NBS proposals and their level of detail, and spatial scale, are used to assess how widely NBS are distributed across the Canary territory and how advanced their design is as an enabler of local adaptation. Each proposal was counted once per unique ecosystem or site, even if mentioned multiple times across the documents. The level of detail (I.2) is scored using a system adapted from Cortinovis and Geneletti (2018) and Geneletti and Zardo (2015), assigning scores from one to four depending on how many of the following elements were included in the proposal’s description (Table 2), including the ecosystem type to create or intervene (e.g., a community garden or a riverbank), location, size (e.g., hectare or explicit spatial reference), and ecological/technical specifications (e.g., vegetation density, soil removal). Proposal scale (I.3) refers to the implementation zone, classified into five levels (Table 3) and adapted from Nature4Cities (2018). Scale was recorded directly from the documents if spatially specified or inferred from the document’s governance level. It is scored from one to five, increasing with scales. Both the level of detail and spatial scale were scored at the proposal level, then averaged by NBS sub-type and normalized to a 0–1 range for comparative purposes.

Scaling-up indicators assess actors’ involvement, diversity and collaboration. I.4 counts the total number of proponents per NBS sub-type. I.5 measures the share of proposals involving more than one actor, either as proponents or developers, implying some level of collaboration. Indicators I.6-I.8 specify the type of collaboration: multi-level (spanning governance levels), cross-sectoral (across policy areas/departments), or both. For each of these, two complementary indicators were used: (a) a proposal-based indicator, which assesses the prevalence of collaboration across proposals for the same NBS type or sub-type, and (b) a collaboration-based indicator, which captures the diversity and structure of collaborative arrangements.

The scaling-deep indicators analyze the multifunctionality of NBS proposals in terms of ecosystem services and/or societal challenges addressed, to reflect how institutional actors perceive and value NBS. Multifunctionality refers to the capacity of NBS to simultaneously deliver multiple ecosystem services and societal benefits (e.g., combining microclimate regulation, biodiversity enhancement, recreation, and social cohesion). Scaling deep relates to the transformation of cultural norms, beliefs, and values that shape how societies relate to nature (Moore et al. 2015). When institutional actors recognize and intentionally plan for multifunctionality, they are implicitly acknowledging that nature holds diverse types of value: not only instrumental (what nature does for people), but also intrinsic (value in itself) and relational (value through people’s relationships with nature and with one another). Thus, embedding multifunctionality into NBS planning may signal a potential shift in institutional culture toward a plural understanding of nature, rather than treating NBS as a single-purpose tool. To assess this, three indicators were defined based on the documented functions for NBS proposals. Indicator I.9 counts the total number of functions assigned to each sub-type. Indicator I.10 measures the share of multifunctional proposals, which means having two or more functions, per NBS sub-types. Indicator I.11 reports the average number of functions in those multifunctional proposals.

## Results

### Selected policy documents

We identified 23 documents (Table 5), developed across seven policy areas spanning regional, insular and municipal governance levels, and classified into five types:

- Plans (14 documents, of which two regional, four insular, and eight municipal), operational documents that outline concrete measures for implementation.
- Strategies (five documents, of which two regional, one insular, and one municipal), goal-oriented documents primarily establishing broad objectives and priorities.
- Risk analysis (two documents at the insular level), assessment documents designed to evaluate hazards and vulnerabilities and propose measures.
- Programme (one document at the regional level), a coordinated, multi-project framework.
- Guideline (one document at the municipal level), a document providing recommendations and best practices.

**Table 5:** List of selected policy documents, related governance levels and main policy areas. *Given its purpose, structure, and content, this document is classified as a strategy. **This cross-cutting document addresses both climate action and forestry/green infrastructure and ecosystem restoration

		Governance levels		
		Municipal	Insular	Regional
Policy areas	Spatial Planning and Territorial Development	General Plan of LPGC Special Protection Plan of Vegueta-Triana Special Protection Plan of La Mayordomia Special Plan of Puerto de Las Palmas Special Plan of San Nicolas	Insular Plan of Gran Canaria	
	Climate Action	Action Plan for Climate Change	Insular Strategy for Climate Change Adaptation Biodiversity Vulnerability and Risks Accounts for Climate Change** Insular Risk Analysis for Climate Change	Climate Change Canary Strategy
	Environmental sustainability & Ecological transition			FEDER Action Programme Canary Energy Strategy 2115–2025 Canary Islands Energy Transition Plan
	Integrated Water Management		Hydrological Plan of Gran Canaria	
	Sustainable Waste Management		Special Territorial Plan for Waste Management	
	Forestry, Green Infrastructure and Ecosystem Restoration	Municipal Strategy for the Urban Landscape—Reminding Trees Municipal Plan of Palms Planting Community Garden Project: toward Sustainability* Municipal Guideline for Community Gardens		Canary Forest Management Plan
	Gender and Social Inclusion	Municipal Plan for Gender Equality	Strategic Plan for Equality	

### Identification of NBS proposals

We identified 445 proposals across 14 NBS sub-types (Table 6), including two general categories, ecosystems, and urban green spaces and vegetation, used when no specific ecosystem was mentioned. This classification of sub-types reflects the content of the case study documents.

**Table 6:** Classification of the NBS types (Table 1) into sub-types, based on the ecosystem targeted by the interventions. Type A includes interventions for creating new ecosystems, type B for enhancing current ecosystems conditions, type C for sustainably managing ecosystems, and type D for protecting ecosystems

NBS sub-types	Type A	Type B	Type C	Type D
Ecosystems (general category)		✓		✓
Urban green spaces and vegetation (general category)	✓	✓		✓
Vacant lots, brownfields and other urban open spaces		✓		✓
Parks	✓	✓		✓
Private gardens				✓
Infiltration-based solutions	✓			
Forest, woodland and Canary palm community	✓	✓	✓	✓
Shrubland and scrubs		✓	✓	✓
Vegetated linear or edge structures (e.g., corridors, barriers, buffers)	✓	✓	✓	✓
Agricultural landscapes (e.g., banana plantations, grazing areas)		✓	✓	✓
Urban agriculture (e.g., community gardens, orchards)	✓		✓	
Hillsides and volcanic areas (e.g., geomorphologically sensitive terrestrial areas such as volcanic cones, lava fields, eroded or unstable slopes in mining sites, and rocky escarpments (e.g., inland cliffs or “riscos”), excluding watercourses and coastal cliffs.)		✓		✓
Water bodies and courses (e.g., ponds, riverbanks, riverbeds, dry or seasonal channels such as ravines)		✓	✓	✓
Coastal ecosystems (e.g., sandy beaches, dune systems, coastal cliffs, shrubland on volcanic platforms, and shallow coastal inlets and bays)	✓	✓	✓	✓

Of the 23 documents analyzed, two (the Canary Energy Strategy and the Gran Canaria Strategic Plan for Equality) contained no NBS proposals. Plans account for most proposals (407), followed by strategies (24), risk analyses (9), programmes (3), and the guideline (2). They are relatively balanced across enhancement (38%), protection (37%), and creation (21%) interventions, with fewer management proposals (4%). Strategies emphasize enhancement (46%), then management (21%) and protection (21%), with creation representing only 8% of proposals. Risk analysis focus on enhancement (44%) and management (33%), while also including creation (11%) and protection (11%) internventions. Programmes address only protection, and the guideline exclusively management.

### Assessing scaling indicators and emerging patterns

#### Scaling out

Table 7 reports the scaling-out indicators for NBS sub-types and types. Enhancement (type B) and protection (type D) spread spatially across multiple ecosystems, while creation (type A) and management (type C) focus on fewer, mainly parks, vegetated linear structures, forests, woodlands, and Canary palm communities.

**Table 7:**
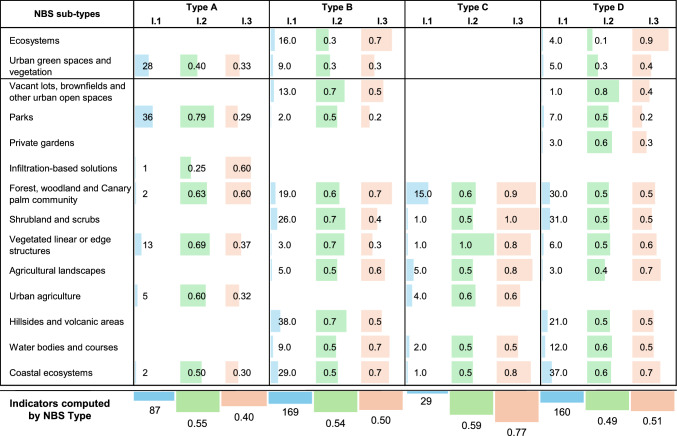
Scaling-out indicators computed by NBS types and sub-types. Indicator codes (I.1–I.3) refer to the definitions provided in Table 4

Four distinct scaling-out patterns emerge. The first, best-performing group shows relatively high proposal counts and moderate-to-high detail and scale. Forests perform consistently across enhancement, management, and protection, while shrublands, hillsides/volcanic areas, and coastal ecosystems mainly under enhancement and protection. The second group has moderate-to-high detail and scale but fewer proposals. It includes enhancement, management and protection in agricultural landscapes and water bodies, enhancement and protection of vacant lots, brownfields and other urban open spaces, management and protection of vegetated linear structures, and creation on new forest patches. The third group features many proposals with moderate-to-high detail but small spatial scale, found only for new urban parks and vegetated linear structures. The fourth group includes NBS sub-types with only one strong indicator—scale (e.g., ecosystem protection/enhancement, new infiltration-based solutions) or detail (e.g., new urban agriculture and coastal ecosystems, protection/enhancement of parks, private gardens, vegetated linear structures)—while the other two indicators remain low. Major gaps appear for private gardens and infiltration-based solutions, where proposals are largely absent.

#### Scaling up

Of 29 actors identified, 26 engaged with NBS as proponents (Table S2 of the Supplementary Material). Most proposals originate from public entities, while academic institutions, consultancies, and private companies play only minor roles.

Proposal distribution varies by governance level, increasing significantly from 35 at regional to 126 at insular, and 284 at municipal level. Creation (type A) is almost entirely municipal (96.6%). Protection (type D) appears mostly in municipal (65.6%) and insular (28.1%) documents. Enhancement (type B) is more balanced (municipal 52.7%, insular 40.8%), while management (type C) is led by regional institutions (48.3%), with fewer insular (31.0%) and municipal (20.7%) proposals.

Twelve collaborations were recorded (Table S3 of the Supplementary Material): two cross-sectoral and seven both multi-level and cross-sectoral. No collaborations were exclusively multi-level (I.6). Most proposals, especially for creation and protection, involved at least two actors, except shrubland management. Yet only 27 proposals qualified as cross-sectoral and/or multi-level. Many collaborations merely linked administrations with consultants, who acted as facilitators commissioned to draft documents rather than independent NBS promoters.

Four distinct scaling-up patterns emerge from Table 8. The most common combines low actor diversity (I.4) with a moderate-to-high share of collaborative proposals (I.5). It spans numerous sub-types, from urban green spaces, vacant lots, and vegetated structures to hillsides, volcanic areas, forests, shrublands, agricultural landscapes, water bodies, and coastal ecosystems. However, only six sub-types show at least one classified collaboration, suggesting collaborations often lack the qualities needed for effective scaling. The second, most frequent pattern shows stronger scaling up, with diverse actors and a high share of collaborative proposals. It appears in five sub-types: ecosystems (enhancement, protection), forests (protection), agricultural landscapes (enhancement), urban agriculture (creation), and water bodies (enhancement). The third pattern combines high actor diversity with weak collaboration, found in forest (enhancement, management) and coastal ecosystems (enhancement, protection), indicating fragmented engagement. The fourth pattern shows low diversity and collaboration, evident in shrubland and urban agriculture management, highlighting a clear gap in collective involvement.

**Table 8:**
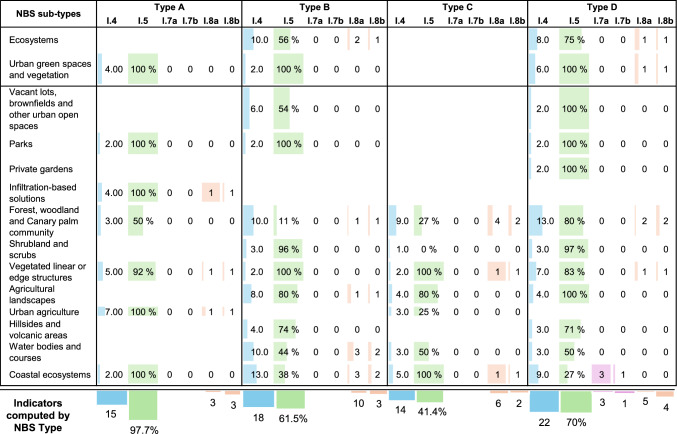
Scaling-up indicators computed by NBS types and sub-types. Indicator codes (I.4–I.8) refer to the definitions provided in Table 4

#### Scaling deep

The revised policy documents link NBS proposals to 50 functions spanning societal challenges and ecosystem services (Table S4).

Results of the scaling-deep indicators (Table 9) reveal seven scaling-deep patterns. The first pattern shows fully integrated multifunctionality in both potential and application, observed in creation of forest patches, woodlands, and Canary palm communities. Specifically, it combines a wide functional range (I.9), a high share of multifunctional proposals (I.10), and strong integration within each (I.11). A second group shows similar commitment but within a narrower scope. Infiltration-based solutions, urban agriculture, and coastal ecosystem creation rely on fewer functions (I.9) yet apply them consistently (I.10, I.11). The third pattern reflects partial integration, found in park creation and forest and shrubland protection. These sub-types cover a broad functional range (I.9) and have a relatively high number of proposals (I.10) but each integrates only a few functions (I.11), suggesting fragmented application. Another case within this pattern shows underutilized potential: urban agriculture management has broad functional range (I.9) and deep integration when applied deep (I.11), yet few proposals use it (I.10). The fourth pattern includes vacant lots and shrubland enhancement, and green space protection, showing basic but consistent multifunctionality: limited functions (I.9), few per proposal (I.11), but steady application across proposals (I.10). The fifth and second most common pattern is underused multifunctionality, found in urban green space creation/enhancement, management of forests, vegetated linear structures, and enhancement/protection of hillsides, volcanic areas, and coastal ecosystems. These sub-types span many functions (I.9) but few proposals are multifunctional (I.10), and those integrate only a small number (I.11). Finally, many sub-types display minimal multifunctionality, including vegetated linear structures and water bodies (enhancement/management/ protection), parks and agricultural landscapes (enhancement/ protection), private gardens and vacant lots (protection), forests enhancement, and shrublands management. These are tied to few functions (I.9) and score low in both frequency (I.10) and depth (I.11).

**Table 9:**
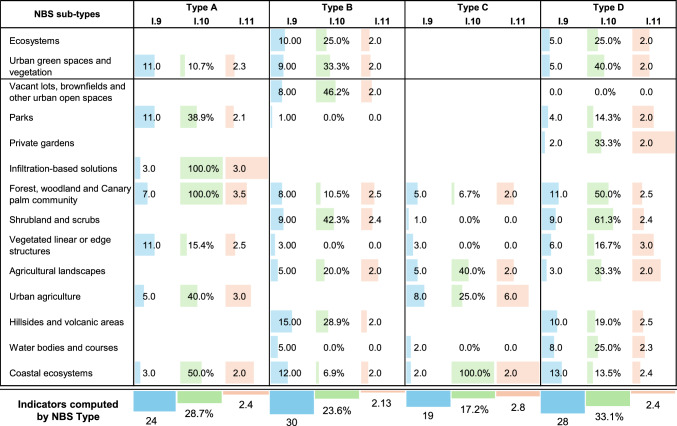
Scaling-deep indicators computed by NBS types and sub-types. Indicator codes (I.9–I.11) refer to the definitions provided in Table 4

### Patterns between scaling strategies across NBS types

**Fig. 1:**
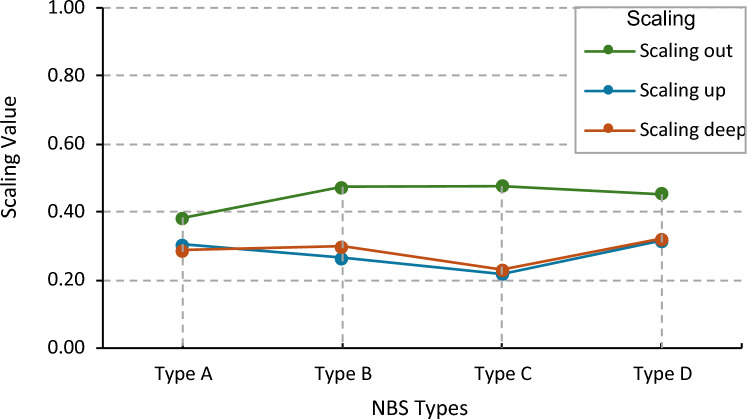
Aggregated performance of scaling indicators across NBS types

Figure 1 compares scaling profiles across NBS types. Protection (type D) stands out with the highest values for scaling up (0.32) and scaling deep (0.32), plus a strong scaling-out score (0.45). Enhancement (type B) shows strong scaling-out value (0.47) but only moderate scaling up (0.27) and deep (0.30), indicating broad adoption with partial institutional and functional support. Creation (type A) is more balanced but slightly lower across strategies (0.38, 0.30, 0.29 for scaling out, up and deep, respectively). Management (type C) performs weakest overall, especially in scaling up (0.22) and deep (0.23); despite a high scaling-out score (0.48).

Overall, scaling out consistently outperforms the other strategies. Three cross-strategy scaling patterns emerge. First, a clear link appears between scaling out (notably indicator I.3) and scaling up: territorial expansion often occurs with limited actor involvement, seen in management interventions, forest patch and infiltration-based solutions creation, and hillside/shrubland enhancement. Second, two patterns link scaling out and scaling deep. One reflects “quantity over quality”, with broad implementation but narrow functional goals, e.g., hillside, volcanic, coastal enhancement/protection and forest management. The other one suggests scaling-deep potential not yet taken to scale: some sub-types show wide functional ranges but with few proposals, such as forest patch creation, landscape and urban agriculture management, coastal ecosystem creation and management, and urban green spaces protection. Third, scaling up and scaling deep interact in two ways. Some cases show high actor diversity or collaboration but weak functional ambition, e.g., enhancement of water bodies, coastal ecosystems, agricultural landscapes. Others show strong multifunctional design without collaborative processes, such as managing urban agriculture.

## Discussion

### NBS scaling in the study area

Overall, NBS in the case study fall short in being systematically mainstreamed spatially, through governance levels and sectors, and anchored to decision-makers’ values. Across all NBS types, scaling out consistently outperforms scaling up and scaling deep, indicating their replication is more advanced than institutional embedding or functional integration.

Protection- and enhancement-focused NBS show stronger integration across spatial, governance, and functional dimensions, likely reflecting the Canary Islands’ status as a biodiversity hotspot and their long-standing preservation and restoration practices. Creation-oriented NBS are promising but still evolving in policy support and functional breadth. In contrast, management interventions perform weakest in scaling up and scaling deep, despite depending most on cross-sectoral, multi-level, and spatial coordination for long-term resilience. Scaling-out–scaling-up patterns suggest these interventions are often imposed through top-down land-use policies lacking local ownership and input.

Scaling out manifested through more projects, larger areas and/or detailed technical prescriptions. Many sub-types, especially in management, combine the latter two aspects, pointing to established planning practices. Yet, some scaling patterns suggest small-scale projects often include more detailed designs, supporting tailored responses to specific local ecosystem service demands.

Scaling-up results for NBS types varies by governance levels: creation and protection are locally anchored, enhancement more evenly shared, and management centrally coordinated. This highlights the potential of municipal and insular actors for bottom-up scaling, echoing Barcelona, where municipal leadership drove NBS adoption despite limited national support. (Kauark-Fontes et al. 2023). In the Canary Islands, municipal councils wield strong authority over land use and infrastructure (Cortes Generales de España 2018), while insular and regional levels mostly provide strategic guidance without binding instruments (Dasí et al. 2005). Moreover, scaling-up patterns show two issues. First, many sub-types show missed opportunities to engage more sectors (and related actors) as creators, enhancers, managers, and protectors of nature. Notably, actors’ involvement beyond public administration is limited. This gap is also reflected in scaling out, where interventions in private gardens are scarce. Aside from a few mentions of private landowners, the economic private sector is largely absent, mainly at the neighborhood level, which is especially suited for private sector involvement as small businesses dominate the local economy (González de la Fe et al. 2012; The World Bank 2021). Second, multi-actor collaborations are rare and mostly confined within single policy areas or governance levels, offering little space for co-creation or knowledge exchange, hence weak capacity to foster the kind of partnerships needed to enable scaling.

Scaling-deep patterns reveal minimal or underused multifunctional potential. Although NBS sub-types reference a broad set of functions, proposals often remain aligned with sectoral priorities, even when institutional support and collaborations exist (as reflected in scaling-up–scaling-deep patterns), overlooking co-benefits as a key feature of NBS. Notably, proposals involving collaboration between multiple actors often lack alignment with multifunctional ambitions, tending to address only a narrow set of objectives rather than delivering a diverse suite of ecosystem services or societal benefits. This suggests that collaboration, while valuable, might not automatically lead to more integrated or holistic NBS planning. Addressing multiple challenges at once signals a shift toward more systemic approaches to the biodiversity-climate-society nexus (Pascual et al. 2022), but this nexus is still missing in the Canarian planning context. Similar limits are noted by Korkou et al. (2023) and Lemes de Oliveira (2025), who observe that while plural values of NBS are increasingly recognized, their integration into planning remains weak, posing a barrier to long-term uptake.

Another scaling-up–scaling-deep pattern, seen in non-collaborative multifunctional proposals, may reflect how technocratic planning, often driven by consultancies or expert teams rather than participatory and co-creation processes, can produce technically robust designs but lack social anchoring. Addressing this is crucial, as people’s perceptions and relationships with nature vary widely (Mattijssen et al. 2020). Finally, some multifunctional proposals scored low on scaling out, remaining conceptually rich but weakly implemented.

### Opportunities for improving NBS scaling

Drawing on case study patterns, this section discusses opportunities, both practical and research-oriented, to reinforce interconnected scaling-out, scaling-up, and scaling-deep strategies.

Policy documents shape the institutional environment for NBS. Each serves complementary roles. Particularly, plans are central in our analysis, ensuring land uses for NBS and enabling vertical coordination through their nested hierarchy from regional to municipal levels, contributing to a coherent, well-connected territorial green infrastructure. Furthermore, their use across diverse policy areas allows NBS to be embedded in the operational logic of multiple sectors beyond the environmental domain. Yet without the narratives of strategies, the legitimacy of risk analyses, the coalitions and funding integration of programmes, and the know-how of guidelines, plans’ transformative potential remains limited.

Effective scaling up requires more than coordinated documents: it depends on inclusive, collaborative processes. From our findings, formal inclusion of actors does not necessarily yield transformative collaborations. Multiple actors’ inclusion must be coupled with co-creation, co-design, and co-production (Cohen-Shacham et al. 2019; Frantzeskaki 2019; Wickenberg et al. 2021; Kabisch et al. 2022), supported by partnerships, advocacy coalitions, and specific enabling conditions. Mabon et al. (2022) highlight the importance of recognizing diverse knowledge systems, such as scientific, technical, and traditional, as equally valid sources of NBS evidence and integrating them to avoid disciplinary silos and reliance on purely quantitative logic that alienate communities. Deep and inclusive engagement must empower innovators, youth, elderly, minorities, activists, and NGOs to shape and sustain NBS interventions (Kiss et al. 2022; Mabon et al. 2022; Adams et al. 2023a). In addition, intermediaries, trust-building actors, and learning-oriented activities are crucial in fostering long-term collaboration and institutional anchoring (Frantzeskaki and Bush 2021; Kiss et al. 2022; Adams et al. 2023b).

Scaling up also depends on greater private sector participation. Despite their potential to stabilize funding (European Investment Bank 2023) and buffer uncertainty and short-term political cycles (Lambin et al. 2020), private actors remain marginal in the case study. Market-based instruments and public–private partnerships are increasingly used to attract private investment (Baroni et al. 2019), often supported by economic valuation methods such as cost–benefit analysis (Chelli et al. 2025a, b). Yet over-reliance on private capital can steer NBS implementation toward projects that prioritize economic returns over ecological or social value (Chausson et al. 2023), risking green gentrification (Anguelovski et al. 2018; Goossens et al. 2020), inequitable access (Thompson et al. 2023), greenwashing practices (Gałecka-Drozda et al. 2021), and the erosion of collective values (Huang et al. 2024). Research is needed on how private motivations might align with multifunctional, biodiversity-positive, and just NBS.

Although NBS inherently deliver multiple ecosystem services, actors often overlook multifunctionality in policy documents. This limits capacity to address pressing challenges or generate impact at scale. Three aspects matter: (i) the spatial arrangement of biotic and abiotic components in NBS shapes services supply (Orta-Ortiz and Geneletti 2022), (ii) not all NBS types deliver all services equally (Babí Almenar et al. 2021), and (iii) some services provisions are scale-dependent in both a spatial (Demuzere et al. 2014) and temporal way (Chelli et al. 2025a, b). For instance, noise reduction requires a minimum area, while air purification, carbon sequestration, and runoff mitigation only materialize beyond certain thresholds (Cortinovis and Geneletti 2019). This has strong implications for designing scaling-out strategies as replicating the same NBS type—sometimes at the expense of local multifunctionality—may be needed to achieve benefits at broader scales (Orta-Ortiz and Geneletti 2023). Hence, more effective scaling out requires contextualizing multifunctionality including defining the spatial and temporal scope of challenges, identifying ecosystem service’s needs, assessing their trade-offs across scales, and fostering dialogue among decision-makers and sectors.

The narrow functional framing found in this study suggests a weak (institutional) cultural embedding of NBS. This, combined with the limited engagement from civil society and non-policy actors, raises concerns about the capacity of spatial planning and policy to integrate diverse ways of knowing, valuing, and relating to nature. Specifically, relational values capture the significance people place on their connection with nature and with others in natural settings (Chan et al. 2016), nurturing care, reciprocity, identity, and sense of place (Schröter et al. 2020; Pratson et al. 2023). Ignoring them reinforces what Pascual et al. (2023) call a “values crisis”, a barrier to effective biodiversity governance. Building on Lemes de Oliveira (2025), scaling deep depends on culturally grounded relationships where people (e.g., institutional actors in our study) see themselves as protectors, creators, managers, and stewards of nature, and reflect these roles in policy documents. Yet a major gap remains in operationalizing these values in spatial planning’s institutional settings and processes, shifting from a purely rational approach to managing natural resources toward one that also prioritizes nature connectedness and non-material benefits (Clark 2011; Roux et al. 2022; Dewi et al. 2023). Some scholars advocate for incorporating plural valuations of nature through integrated ES assessment methods, fostering relational language in policy, and reoriented education (Schulz and Martin-Ortega 2018; Mattijssen et al. 2020; Dewi et al. 2023; Raymond et al. 2023).

### Limitations of the study

The proposed indicators provide valuable insights into scaling-out, scaling-up, and scaling-deep strategies of NBS. Disaggregated results by NBS type clarify why some interventions align more naturally with specific scaling pathways. Yet the analysis, based solely on policy documents, cannot capture the full complexity of scaling dynamics. First, it reflects what is stated *in* documents, not what is implemented. Thus, it cannot assess whether proposals translate into tangible landscape or ecosystem changes, which is crucial for evaluating scaling-out success. Future research should complement document analysis with spatial data (e.g., satellite imagery, field surveys) to validate implementation and biodiversity outcomes. Second, NBS sub-type classification was developed qualitatively and tailored to the local ecological and governance context. While consistent with broader typologies, this may limit replicability and comparability across regions. Third, although multiple actors are identified through document authorship or acknowledgments, the study does not assess the depth or nature of their participation. Understanding actors’ roles and decision power requires more in-depth qualitative methods such as targeted interviews and surveys.

## Conclusion

This paper refines the definitions of scaling out, scaling up, and scaling deep for NBS and introduces measurable indicators for each. Applied to multi-level policy documents from the Canary Islands, these indicators show how spatial, institutional, and cultural dimensions of scaling shape the uptake of NBS.

Findings reveal a range of contrasting scaling patterns. These include frequent small-scale projects tailored to local needs versus fewer, large-scale initiative with greater technical detail. Some cases show broad implementation without sufficient actor engagement, while others point to untapped opportunities for cross-sectoral collaboration and bottom-up leadership. Finally, some proposals exhibit minimal or underused multifunctionality, whereas others are functionally rich but not collaborative, or highly collaborative yet limited in functional ambition.

While there is no panacea for scaling NBS, alignment between strategies is essential. Scaling up, via broader actor engagement, stronger collaboration, and coordinated policy documents, can create enabling conditions for more effective scaling out. Without strong institutional anchoring and shared responsibility, spatial implementation risks becoming fragmented. At the same time, scaling out must go beyond expanding number and size of NBS interventions; it should embed scaling deep by integrating multifunctionality into planning and design. This requires stronger institutional recognition, incorporating diverse (particularly relational) values of nature, seeing it not only as a resource but as something people relate to, care and take responsibility for.

## Supplementary Information

Below is the link to the electronic supplementary material.

Supplementary file1 (PDF 779 KB)

## Data Availability

Coded text fragments extracted from the reviewed policy documents are available from the authors upon reasonable request.
