# Supplementary material for: Assessing scaling strategies for nature-based solutions: An indicator-based analysis of policy documents in the Canary Islands
Source: Ambio. 2026 Apr 12;55(9):2250–65. doi: 10.1007/s13280-026-02360-8 (PMC13437867; doi:10.1007/s13280-026-02360-8)
Supplement: Supplementary file 1 [file 13280_2026_2360_MOESM1_ESM.pdf]

Supplementary Material of the paper entitled “Assessing Scaling Strategies for Nature-based Solutions: An Indicator-based Analysis of Policy Documents in the Canary Islands”

Table S1: List of keywords used to identify NBS proposals in policy and planning documents. A translation in Spanish was entered in MAXQDA software.

**List of keywords**	
Vacant space, or site	Protected area
Beach	Landscape
Park	Tree
Green space	Vegetation
Grove	Conservation
Pond	Regeneration
Stream	Reforestation
Gardening or garden	Rehabilitation
Green corridor	Renaturalization
Transition area	Protection
Wooded or forested area	Restoration
Forested area	Ecological improvement
Natural habitat	Planting
Ecosystem	

Table S2: List of actors involved in NBS proposals.

Governance level	Public administrations and agencies	University, Research Institutions, Consultancies
National	State Meteorological Agency<br>Biodiversity Foundation, Ministry of Agriculture, Fisheries, Food, and Environment<br>Port Authority of Las Palmas	
Regional	Department of Ecological Transition, Climate Change Mitigation, and Territorial Planning<br>Deputy Department for Climate Change Mitigation<br>General Directorate for Climate Change Mitigation and Environment<br>General Directorate for Energy<br>Biodiversity Service	Canary Islands Technological Institute<br>GESPLAN - Territorial and Environmental Management and Planning, S.A.
Insular	Gran Canaria Island Council<br>Department of Territorial Policy and Landscape<br>Department of Environment<br>Gran Canaria Island Energy Council<br>Gran Canaria Island Water Council	Grupo Considera S.L.
Municipal	Department of Urban Planning	University of Las Palmas de Gran Canaria

	Department of Mobility and Environment<br>Department of Tourism, Employment, and Local Development<br>Department of Equality<br>Department of Public Services and Carnival*<br>Municipal Services for Parks, Gardens, Environment, and Urban Planning<br>Local Energy Management Agency	GEURSA – Urban Planning and Development Management Company, S.A.<br>Socioanálisis, S.L.<br>GIPIC - Integrated Project and Investment Management in the Canary Islands, S.L.

**Actor mentioned as developer.*

Table S3: List and type of actors' collaborations

Actors' collaborations	Collaboration types	
	Cross-sectoral	Cross-sectoral & multi-level
Department of Ecological Transition, Climate Change Mitigation, and Territorial Planning;<br>GESPLAN - Territorial and Environmental Management and Planning, S.A.	n.a.	n.a.
Department of Ecological Transition, Climate Change Mitigation, and Territorial Planning;<br>GESPLAN - Territorial and Environmental Management and Planning, S.A.;<br>Deputy Department for Climate Change Mitigation;<br>General Directorate for Climate Change Mitigation and Environment;<br>Biodiversity Service	✓	
Department of Equality;<br>Socioanálisis, S.L.	n.a.	n.a.
Department of Tourism, Employment, and Local Development;<br>Gran Canaria Island Energy Council;<br>Local Energy Management Agency;<br>Canary Islands Technological Institute		✓
Department of Tourism, Employment, and Local Development;<br>Gran Canaria Island Energy Council;<br>Local Energy Management Agency;<br>Canary Islands Technological Institute<br>Department of Urban Planning		✓
Department of Tourism, Employment, and Local Development;<br>Gran Canaria Island Energy Council;		✓

Local Energy Management Agency;<br>Canary Islands Technological Institute<br>Department of Public Services and Carnival		
Department of Urban Planning;<br>GEURSA – Urban Planning and Development Management Company	n.a.	n.a.
Department of Urban Planning;<br>Port Authority of Las Palmas;<br>GIPIC - Integrated Project and Investment Management in the Canary Islands, S.L		✓
General Directorate for Energy;<br>Canary Islands Technological Institute	✓	
Gran Canaria Island Council;<br>Grupo Considera S.L.;<br>State Meteorological Agency;<br>Canary Islands Technological Institute;<br>University of Las Palmas de Gran Canaria<br>Local entities		✓
Gran Canaria Island Energy Council;<br>Biodiversity Foundation		✓
Gran Canaria Island Water Council<br>Gran Canaria Island Energy Council;<br>Biodiversity Foundation		✓

Table S4: List of functions associated with NBS proposals in policy documents.

Functions	Number of proposals
**Societal challenges** (extracted from Raymond et al. (2017) and Babí Almenar et al. (2021))	
**Quality of life**, i.e., enhance overall quality of life in urban areas.	1
**Public participation**, i.e., enhance the inclusion of citizens, private/semi-private/public associations and NGO’s in the decision-making.	1
**Education**, i.e., support environmental knowledge transfer between generations.	2
**Gender disparities**, i.e., reduce gender inequity regarding access to any type of urban services.	1
**Urban cohesion**, i.e., address the lack of cohesion in the morphological and functional structure of territories represented by a lack of connectivity, segregation and marginalization, lack of functions, and loss of socio-economic dynamics.	9
**Built Environment**, i.e., enhance the planning, development and management of built physical structures.	2

**Urban transition**, i.e., ensure transition between contrasting elements in urban-rural areas	12
**Lack of liveable and adaptable public spaces**, i.e., mitigate the lack of multifunctional and active public spaces in urban areas that could jeopardise socio-economic activities and the use of public spaces by inhabitants.	6
**Urban, industrial and agricultural sprawl**, i.e., limit and counteract the impacts of the excessive and inadequate expansion of urban areas, and other land uses.	46
**Ageing and inadequate infrastructure**, i.e., mitigate or overcome the ageing and inadequate planning and management of infrastructures.	3
**Landscape quality**, i.e., address the landscape degradation (e.g., abandoned lots, loss of traditional features) and enhance the landscape visual quality.	71
**Cultural heritage and identity**, i.e., preserve and enhance the built cultural heritage and the elements (tangible or not) that form part of the cultural identity.	1
**Sustainable agriculture**, i.e., promote agriculture practices based on sustainability principles.	1
**Tourism activities**, i.e., support tourism activities by preserving the natural attractiveness of sites.	1
**Environmental impact**, i.e., mitigate overall environmental impacts and degradation of human activities and infrastructures	55
**Traffic impacts**, i.e., mitigate environmental impacts caused by transport systems	8
**Waste discharge**, i.e., prevent and restore natural areas degraded due to waste discharge activities.	11
**Mining activities**, i.e., prevent and mitigate the environmental impacts of mining activities.	8
**Air quality**, i.e., improve the air quality in the urban area by planning and management of its physical spaces and urban activities.	1
**Climate Change**, i.e., mitigate or adapt to the change of climate.	5
**Water shortage**, i.e., prevent and increase resilience against water scarcity periods.	1
**Extreme rainfall events**, i.e., enhance the hydrologic response against intensive and frequent rainfall events.	1
**Sea level rise and coastal resilience**, i.e., adapt to modification of sea levels and coastal water behaviors affecting current coastal functions.	3
**Flooding risk**, i.e., reduce the risk of flooding and the associated socio-economic impacts.	1
**Biodiversity**, i.e., mitigate the loss and decline of species population.	76
**Habitat loss and degradation,** i.e., mitigate the reduction and degradation of habitats and ensure continuity of ongoing restorative processes.	55
**Soil quality**, i.e., address the loss of soil properties.	6
**Ecological integrity and connectivity**, i.e., mitigate the loss of naturalness and complexity of habitats and ensure their connectivity.	7
**Ecosystem Services**	

Enhance overall provision of ecosystem services.	5
**Provisioning**	
Cultivated terrestrial plants (including fungi, algae) grown for nutritional purposes	1
Wild animals (terrestrial and aquatic) used for nutritional purposes	1
Surface water used as a material (non-drinking purposes)	1
**Regulation & Maintenance**	
Bio-remediation by micro-organisms, algae, plants, and animals	1
Filtration/sequestration/storage/accumulation by micro-organisms, algae, plants, and animals	1
Noise attenuation	2
Visual screening	1
Control of erosion rates	8
Buffering and attenuation of mass movement	1
Hydrological cycle and water flow regulation (Including flood control, and coastal protection)	5
Fire protection	16
Pest control (including invasive species)	3
Regulation of chemical composition of atmosphere and oceans	3
Regulation of temperature and humidity, including ventilation and transpiration	1
**Cultural**	
Characteristics of living systems that that enable activities promoting health, recuperation or enjoyment through active or immersive interactions	35
Characteristics of living systems that enable activities promoting health, recuperation or enjoyment through passive or observational interactions	36
Characteristics of living systems that enable scientific investigation or the creation of traditional ecological knowledge	1
Characteristics of living systems that enable education and training	2
Characteristics of living systems that are resonant in terms of culture or heritage	5
Characteristics of living systems that enable aesthetic experiences	5
Characteristics or features of living systems that have an existence value	3

References

Babí Almenar, Javier, Thomas Elliot, Benedetto Rugani, Bodénan Philippe, Tomas Navarrete Gutierrez, Guido Sonnemann, and Davide Geneletti. 2021. "Nexus between Nature-Based Solutions, Ecosystem Services and Urban Challenges." *Land Use Policy* 100(April 2019):104898.

Raymond, Christopher M., Berry Pam, Margaretha Breil, Mihai R. Nita, Nadja Kabisch, Mark de Bel, Vera Enzi, Niki Frantzeskaki, Davide Geneletti, Marco Cardinaletti, Leor Lovinger, Corina Basnou, Ana Monteiro, Holger Robrecht, Gregorio Sgrigna, Laura Munari, and Carlo Calfapietra. 2017. *An Impact Evaluation Framework to Support Planning and Evaluation of Nature-Based Solutions Projects. Report Prepared by the EKLIPSE Expert Working Group on Nature-Based Solutions to Promote Climate Resilience in Urban Areas*.
